# Background enhancement in contrast-enhanced spectral mammography (CESM): are there qualitative and quantitative differences between imaging systems?

**DOI:** 10.1007/s00330-022-09238-9

**Published:** 2022-12-06

**Authors:** Daniel Wessling, Simon Männlin, Ricarda Schwarz, Florian Hagen, Andreas Brendlin, Susann-Cathrin Olthof, Valerie Hattermann, Sebastian Gassenmaier, Judith Herrmann, Heike Preibsch

**Affiliations:** Department of Diagnostic and Interventional Radiology, University Hospital of Tuebingen, Hoppe-Seyler-Strasse 3, 72076 Tuebingen, Germany

**Keywords:** Mammography, Digital mammography, Female, Breast density, Menstrual cycle

## Abstract

**Objective:**

To evaluate the impact of the digital mammography imaging system on overall background enhancement on recombined contrast-enhanced spectral mammography (CESM) images, the overall background enhancement of two different mammography systems was compared.

**Methods:**

In a retrospective single-center study, CESM images of *n* = 129 female patients who underwent CESM between 2016 and 2019 were analyzed independently by two radiologists. Two mammography machines of different manufacturers were compared qualitatively using a Likert-scale from 1 (minimal) to 4 (marked overall background enhancement) and quantitatively by placing a region of interest and measuring the intensity enhancement. Lesion conspicuity was analyzed using a Likert-scale from 1 (lesion not reliably distinguishable) to 5 (excellent lesion conspicuity). A multivariate regression was performed to test for potential biases on the quantitative results.

**Results:**

Significant differences in qualitative background enhancement measurements between machines A and B were observed for both readers (*p* = 0.003 and *p* < 0.001). The quantitative evaluation showed significant differences in background enhancement with an average difference of 75.69 (99%-CI [74.37, 77.02]; *p* < 0.001). Lesion conspicuity was better for machine A for the first and second reader respectively (*p* = 0.009 and *p* < 0.001). The factor machine was the only influencing factor (*p* < 0.001). The factors contrast agent, breast density, age, and menstrual cycle could be excluded as potential biases.

**Conclusion:**

Mammography machines seem to significantly influence overall background enhancement qualitatively and quantitatively; thus, an impact on diagnostic accuracy appears possible.

**Key Points:**

*• Overall background enhancement on CESM differs between different vendors qualitatively and quantitatively.*

*• Our retrospective single-center study showed consistent results of the qualitative and quantitative data analysis of overall background enhancement.*

*• Lesion conspicuity is higher in cases of lower background enhancement on CESM.*

**Supplementary Information:**

The online version contains supplementary material available at 10.1007/s00330-022-09238-9.

## Introduction

Contrast-enhanced spectral mammography (CESM) is an imaging technique that was first performed in 2003 [1, 2]. Before image acquisition, patients receive an iodine-based contrast agent intravenously (1.5 mL/kg body weight) [1, 3]. Using the dual energy technique, one image at a low energy level (26–33 kVp) and one at a high energy level (44–50 kVp) are obtained [4]. While the low energy level image resembles a normal digital mammogram, the high energy level image additionally shows areas with iodine uptake, but cannot be used for diagnostic purposes [5]. Digital subtraction of both images creates a recombined image of the breast of diagnostic quality showing the uptake of contrast media [3, 6, 7]. Breast cancer can be detected on CESM due to its neoangiogenesis with concomitant iodine uptake [8–10]. By means of an intense microvascularisation and vascular density, higher than in normal breast tissue, breast cancers have a higher accumulation of contrast medium than breast parenchyma or normal intramammary vessels [11]. The dual-energy technique allows a differentiation between areas with high and those with low contrast medium absorption through different k-edge X-ray absorptions. Areas with low contrast medium uptake are to be eliminated by subtraction.

In previous studies, CESM has shown high sensitivity and specificity in the detection of breast malignancies [12, 13], being mostly still inferior to breast magnetic resonance imaging (MRI) in terms of diagnostic accuracy [14, 15]. High breast density is associated with an increased risk of breast cancer [16]. At the same time, a high breast density increases the probability of a higher parenchymal background enhancement in CESM [17, 18]. The informative value of conventional mammography is limited for these patients. The advantage of CESM is that glandular parenchyma can be suppressed by the dual-energy technique, which enables greater sensitivity in early detection of suspicious findings [19]. Consequently, it is important that a small background enhancement can be guaranteed for these patients.

Similar to MRI, in CESM, there is a certain background enhancement of the benign breast parenchyma, even if the date of examination is timed to the menstrual cycle.

In both imaging modalities, CESM and breast MRI, a high level of background enhancement, can influence the radiological qualitative analysis and interpretation and thereby diagnostic accuracy. Thus, it is associated with higher rates of false positive or false negative findings and therefore, a lower sensitivity and specificity [18, 20, 21]. Differences in the mammography machines and the image post-processing of different vendors might impact background enhancement. The construction of machines differs between the vendors. Used anode/filter combinations, the film-focus-distance, and the running grid of both can be different [22]. Thus, a certain impact on background enhancement is conceivable. To the best of our knowledge, there are no studies evaluating the impact of the mammography machine on background enhancement. Background enhancement in CESM can be analyzed quantitatively by placing a ROI [23, 24] and qualitatively by an image evaluation performed by a radiologist, usually on a numbered scale from 1 (minimal) to 4 (marked) [25].

The aim of our study was to analyze background enhancement on CESM images comparing two different mammography machines qualitatively and quantitatively.

## Material and methods

Institutional review board approval was obtained for this single-center study and informed consent was waived due to its retrospective nature (No. 159/2020 BO2). The study followed the regulations of the declaration of Helsinki.

### Inclusion criteria

From January 2016 to September 2019, a total of *n* = 129 female patients who underwent clinically indicated CESM were retrospectively included in our study. All subjects were at least 18 years old and not pregnant or lactating. Sixty-seven patients had suspicious findings (BI-RADS 4 or 5), identified by mammography, B-mode ultrasound, or both. The other patients had unclear findings that needed further evaluation with contrast enhanced imaging and breast MRI could not be performed, or received the examination as part of a high-risk screening, when there were contraindications to obtain breast MRI.

### Imaging technique

CESM images were acquired by using Senographe Essential^TM^ (GE Healthcare), corresponding to machine A and Selenia ® Dimensions ® (Hologic), corresponding to machine B. Both mammography machines were used simultaneously in this time period in our radiology department. All patients received 1.5 ml/kg body weight iodine-based contrast agents, injected intravenously with a flow rate of 3ml per second. One hundred five patients received iopromid (Ultravist® 300, Bayer), 22 patients iomeprol (Imeron® 350, Bracco). For two patients, the applied contrast agent was not documented. The first image was obtained 2 min after the application of the contrast agents; all images were acquired within less than 5 min. Images of machine A were acquired with 22–49 kV while images with image B were acquired with 20–49 kV. In premenopausal patients, the time of examination was adapted to the menstrual cycle, and images were acquired between the 7th and 14th day of the menstrual cycle. If a timing was not possible (e.g., due to hysterectomy, contraceptives or irregular menstrual cycle), no progesterone levels were determined and the examination was performed without an exact timing. In case of a suspicion of cancer or biopsy-proven breast cancer, a timing of the examination to the menstrual cycle was not performed.

### Image analysis

For image evaluation, a dedicated workstation (Centricity RA1000, General Electric) was used. Firstly, all images were analyzed qualitatively by two radiologists with a minimum of 2 years of experience in breast imaging and afterwards verified by one senior radiologist with 10 years of experience in breast imaging. Images were categorized by an overall background enhancement by using a numbered scale of 1 = minimal, 2 = mild, 3 = moderate, and 4 = marked [25]. In event of a deviation, a consensus reading had to be performed. The qualitative analysis also considered the extent to which the suspicious findings could be delimited as such on the basis of the contrast medium uptake. In the case of poor assessability, a higher level of background enhancement was chosen in cases of doubt. Secondly, quantitative image analysis was performed by placing an oval shaped ROI into the image, inspired by the methodology of a recent published study [7]. The ROI was set to include the most applicable extent of the breast. The correct assessment of the ROI was confirmed by a senior radiologist. The ROI results show a dimensionless value that measures and thus quantifies the intensity enhancement within the acquired image averaged over the surface. Each ROI had a minimum size of 3 cm^2^. The assessment of the ROI was performed under supervision of a senior radiologist with 10 years of experience in breast imaging to guarantee the exclusion of pathological findings and artifacts. The artifacts in CESM images can create a brighter, but also a darker image impression. Air trapping artifacts, motion artifacts, and negative contrast enhancement lead to a dark image impression, for example because in air trapping artifacts the contact between the breast tissue and the detector is incomplete, whereas rim artifacts produce an arc-shaped subcutaneous area with a brighter image impression [26, 27]. These differences in brightness are measurable within the ROI and would thus distort the measured mean values. Therefore, known image artifacts and suspicious findings were not included in the ROI. Each ROI was placed three times and the average values of maximum, minimum, mean, and standard deviation were determined within the ROI, as described previously [7, 28].

### Statistical analysis

Statistical analysis was performed using statistical programs (MedCalc Statistical Software version 18.10 (MedCalc Software bvba, http://www.medcalc.org; 2018) and jmp15, MP®, Version *15* SAS Institute Inc1989–2019.). Quantitative data were tested for normal distribution using the Kolmogorov-Smirnov test.

A Mann-Whitney *U* test was performed to compare non-normally distributed data. C Interrater-reliability of ordinal data was tested using Cohen’s kappa with values ≤ 0 as indicating no agreement and 0.01–0.20 as none to slight, 0.21–0.40 as fair, 0.41–0.60 as moderate, 0.61–0.80 as substantial, and 0.81–1.00 as almost perfect agreement [29].

Interrater agreement for quantitative data was tested using the intraclass correlation coefficient (ICC) with values ≤ 0.5 indicating a poor, 0.5–0.75 a moderate, 0.75–0.90 a good, and > 0.9 an excellent agreement [30]. Hodges Lehmann estimation was performed to test for average differences.

We performed a multivariate regression analysis to test for potential biases influencing background enhancement. The factors machine, contrast agent, breast density (ACR A-D), age, and menopausal status were included in the analysis. The menopausal status was analyzed using a Likert scale from 1 to 5 (1, premenopausal; 2, postmenopausal; 3, examination not adapted to the menstrual cycle; 4, irregular menstrual cycle or no menstruation due to contraception).

To test whether the results could have a potential impact on diagnostic accuracy, all images with histologically confirmed pathological findings were assessed for lesion conspicuity. CESM images were rated using a Likert scale from 1 to 5 (1 pathology not reliably distinguishable from background enhancement; 2, poor lesion conspicuity; 3, moderate lesion conspicuity; 4, good lesion conspicuity; 5 excellent lesion conspicuity). The Mann-Whitney *U* test was used to test the results between both machines for differences.

## Results

### Patient cohort and characteristics

**Fig. 1:**
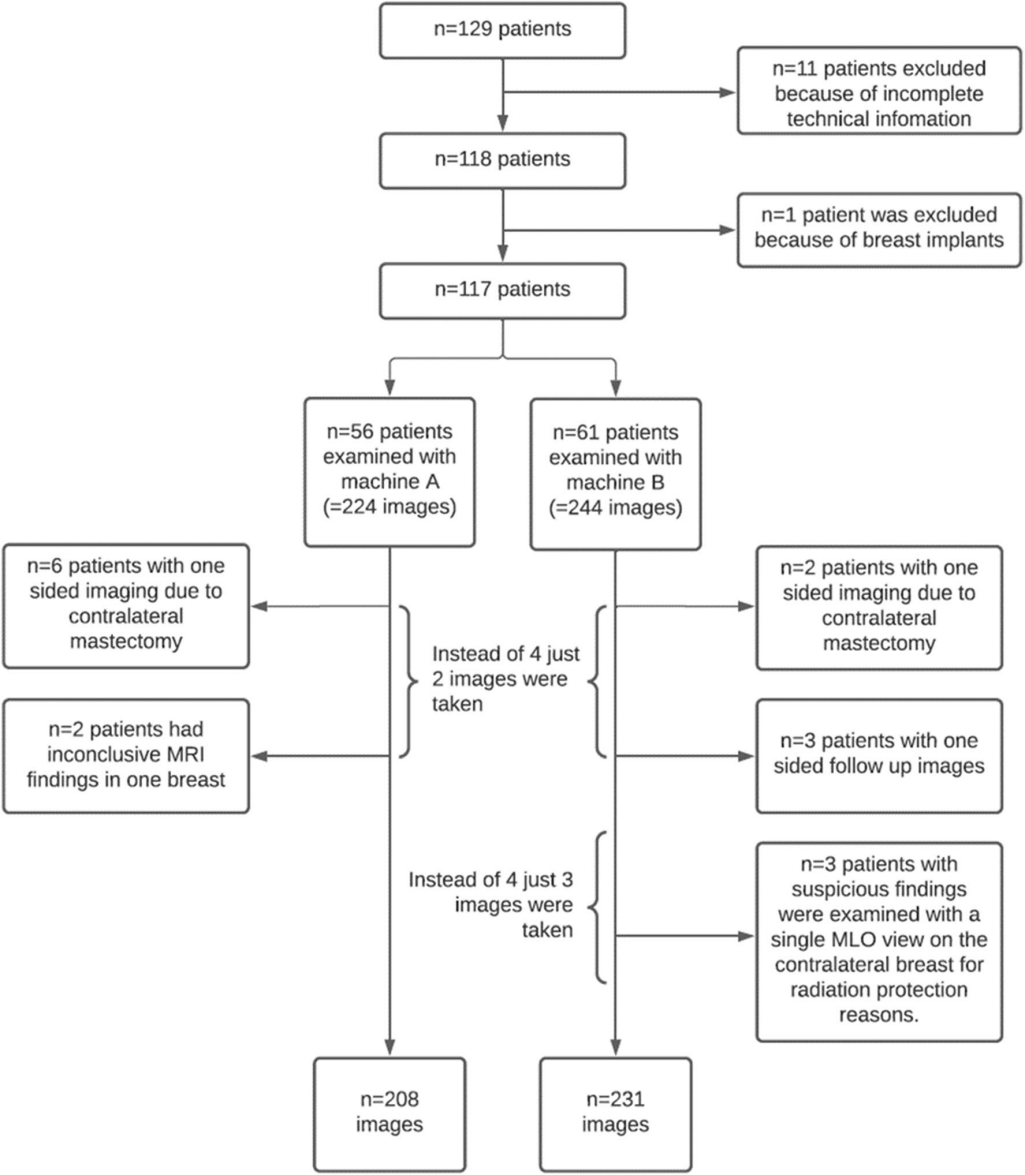
Graphical representation of the exclusion criteria and resulting number of patients and images

One hundred twenty-nine female patients with a mean age of 58 years ± 13.5 (range 30–89 years) were included in the study. *N* = 12 patients had to be subsequently excluded. *N* = 11 subjects were excluded because of incomplete technical information. One patient with breast implants was excluded as this may impair the image quality and reduces comparability. Consequently, the images of *n* = 117 patients were analyzed. In 13 of the 117 patients, only a single-sided image was taken because of contralateral mastectomy (*n* = 8), one-sided follow-up control hy (*n* = 3) or CESM as an additional examination to MRI because of inconclusive findings (*n* = 2). In *n* = 3 patients, imaging was performed in one plane only for radiation protection reasons. This results in *n* = 439 single images. Sixty-one patients were examined with machine A; in 56 cases, the images were taken with machine B. In summary, *n* = 210 single images were acquired by machine A and *n* = 229 images were taken by machine B. An overview of the exclusion criteria and the resulting number of patients and images is shown in Fig. 1.

*N* = 32 patients were premenopausal and had a regular menstrual cycle so that the examination could be timed adequately. *N* = 76 patients were postmenopausal. In *n* = 2 patients, the examination was not timed to the menstrual cycle to avoid therapy delay. *N* = 5 patients had an irregular menstrual cycle so that an adequate timing was not possible.

Detailed patient characteristics of menstrual status and breast density are listed in Table 1.

**Table 1:** Menstrual cycle and breast density according to ACR BI-RADS® classification, distribution by mammography device used

Machine A	Menopausal Status			Breast density		
		Count	Probability	Category	Count	Probability
	Premenopausal	16	0.286	A	0	0
	Postmenopausal	36	0.643	B	21	0.375
	No timing to MC	2	0.036	C	31	0.554
	Irregular MC	2	0.036	D	4	0.071
	Total	56	1	Total	56	1
Machine B		Count	Probability	Category	Count	Probability
	Premenopausal	16	0.262	A	2	0.033
	Postmenopausal	40	0.656	B	31	0.508
	No timing to MC	0	0	C	21	0.344
	Irregular MC	5	0.082	D	7	0.115
	Total	61	1	Total	61	1

*MC* menstrual cycle

### Qualitative analysis

For the images acquired with machine A, a total number of *n* = 104 images were evaluated, whereas *n* = 117 images were acquired with machine B. There was no relevant deviation in the qualitative reading of both readers so that consensus reading was not necessary. The qualitative analysis of the background enhancement of machine A showed a median of 2 (IQR 1–2) for both the first and second readers. The qualitative evaluation of the background enhancement of *n* = 117 images acquired with machine B showed a median value of 2 (IQR 1–3) for both readers.

The detailed results of both readers and their proportional distribution are listed in Tables 2 and 3.

**Table 2:** Results of the qualitative analysis of both readers for machine A

Level of BE	R1 count	R1 percentage	R2 count	R2 percentage
1	44	0.423	43	0.413
2	46	0.442	46	0.442
3	13	0.125	14	0.135
4	1	0.009	1	0.009
Total	104	1.000	104	1.000
Median (IQR)		2 (1–2)		2 (1–2)

*BE* background enhancement, *R1* reader 1, *R2* reader 2, *IQR* interquartile range

**Table 3:** Results of the qualitative analysis of both readers for machine B

Level of BE	R1 count	R1 percentage	R2 count	R2 percentage
1	24	0.205	19	0.162
2	52	0.444	54	0.462
3	23	0.196	27	0.231
4	18	0.154	17	0.145
Total	117	1.000	117	1.000
Median (IQR)		2 (1–3)		2 (1–3)

*BE* background enhancement, *R1* reader 1, *R2* reader 2

**Fig. 2:**
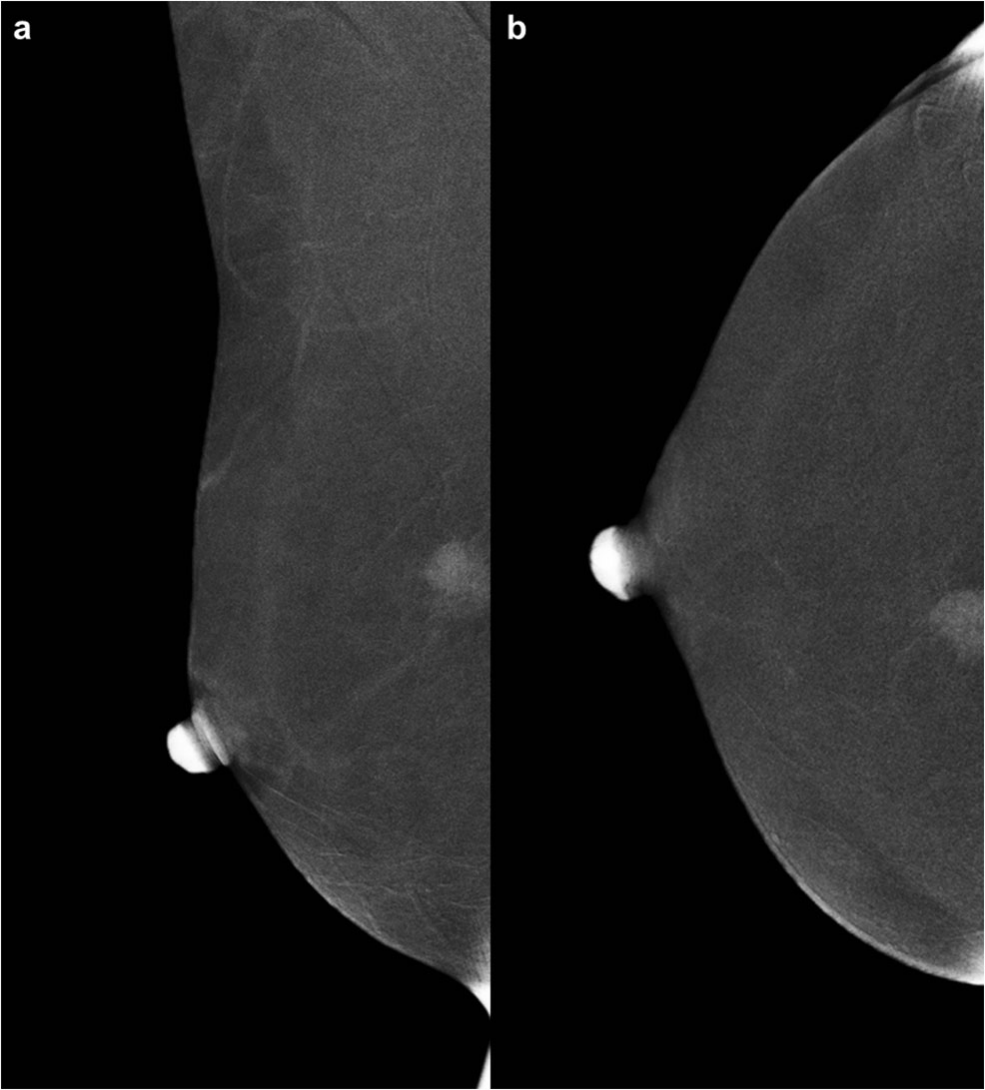
Biopsy-proven invasive lobular carcinoma of a 45-year-olf patient in mediolateral oblique v (**a**) and a craniocaudal view of CESM images (**b**). The images were evaluated by both readers with a background enhancement of level 2 = mild overall background enhancement. Images were taken with machine A

**Fig. 3:**
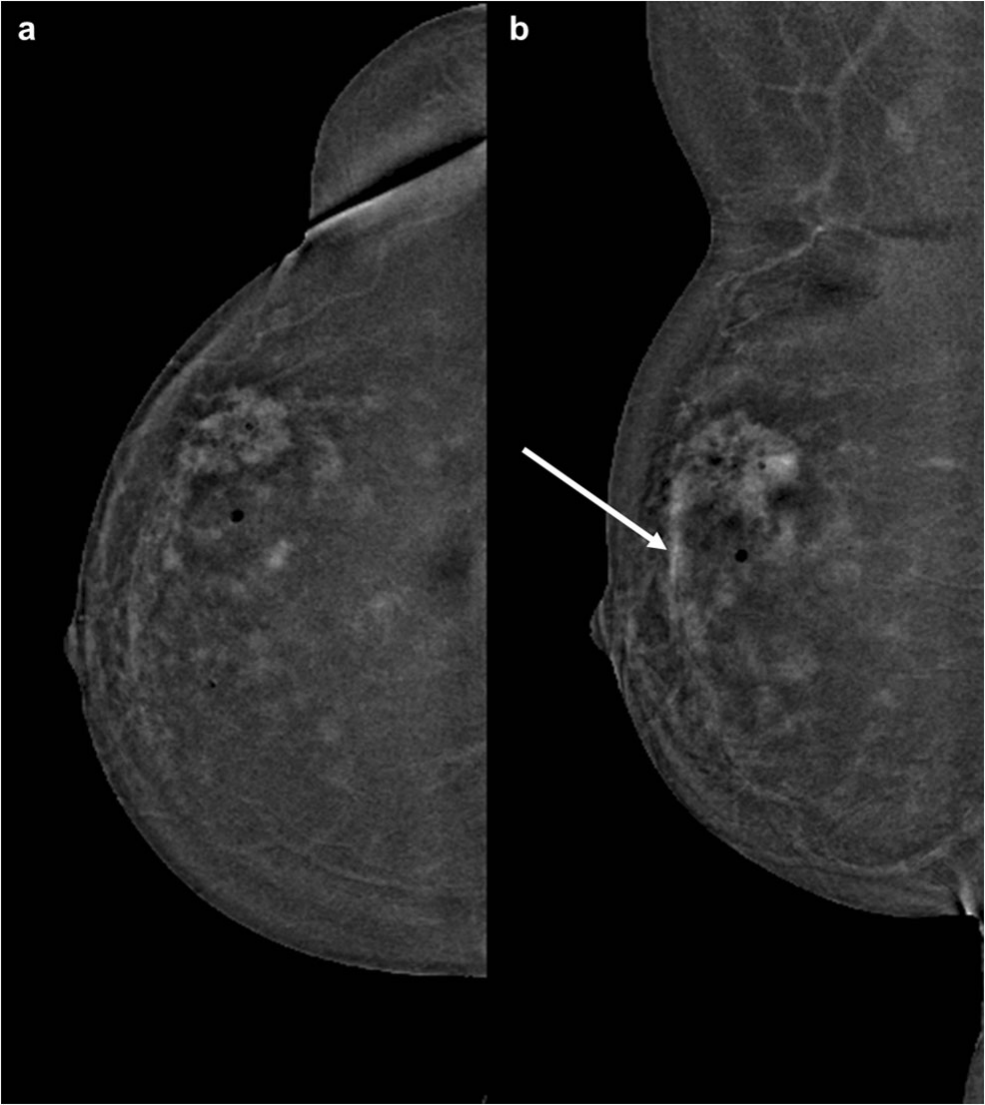
CESM images of a 56-year-old woman with histopathological proven breast cancer in the upper outer quadrant of the right breast. Images were acquired with machine (**b**). Although the malignancy can be clearly detected, the diffuse surrounding enhancement makes the detection of possible satellite lesions and a possible extension in the direction of the mammilla (arrow) almost impossible. The images were rated as showing a marked background enhancement (4/4)

**Fig. 4:**
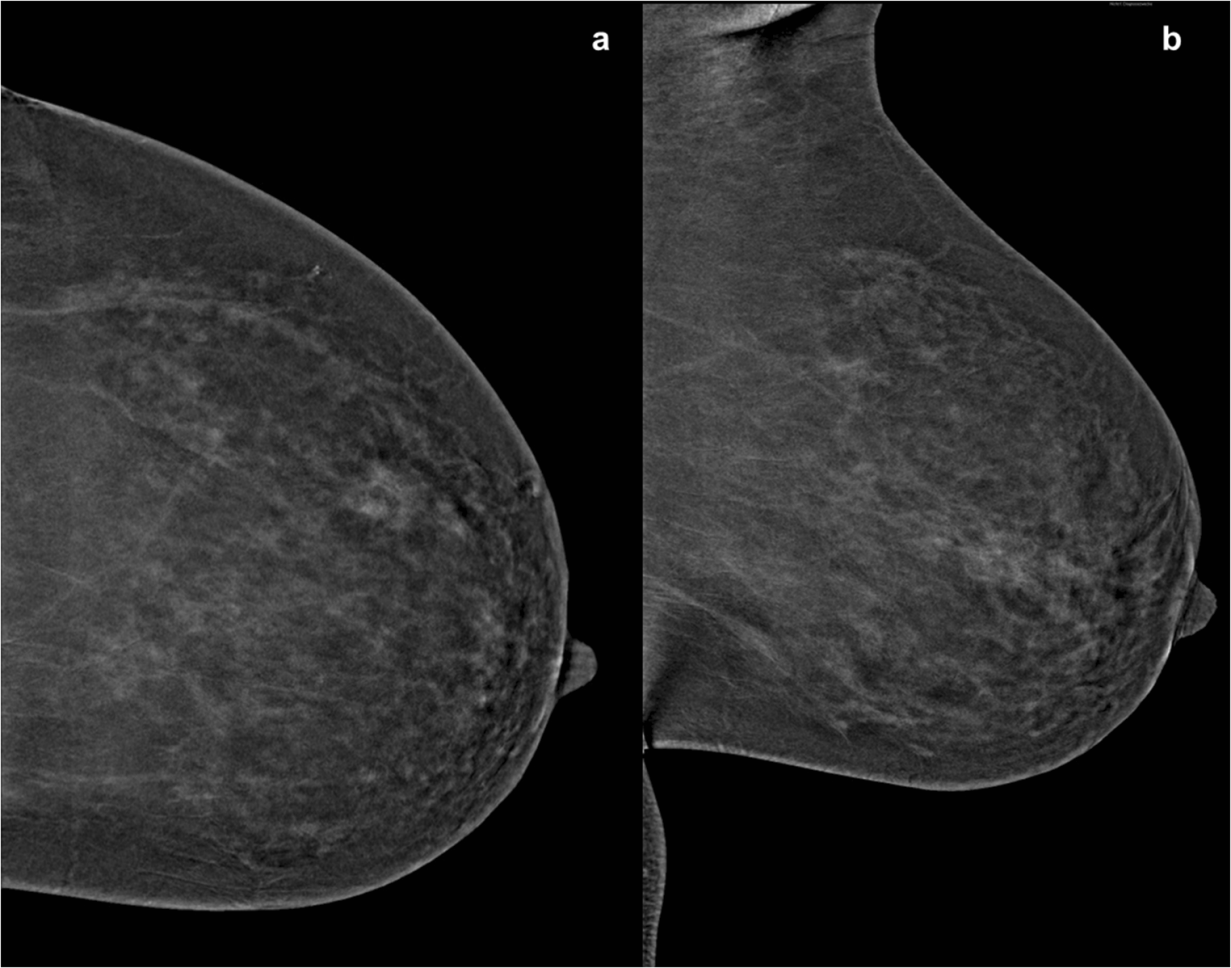
CESM images of the left breast obtained with machine (**b**). The overall background enhancement was rated as 3 (moderate) by both readers in CC (left half) and MLO (right half) view. Hence, it is difficult to detect the exact extent of the suspicious finding

The comparison of the qualitative values of the background enhancement showed statistically significant differences between machine A and machine for both readers (each *p* < 0.001). There was a very strong positive correlation (*p* = 0.885) between the ratings of each reader in both machines. Interrater reliability showed an almost perfect agreement for the images of machine A (0.845) and a substantial agreement for the images acquired with mammography machine B (0.655). The qualitative results showed significant differences between machine A and machine B for both readers (*p* < 0.001). Imaging examples are pictured in Figs. 2, 3, 4.

### Quantitative analysis

The quantitative image analysis was performed using a ROI placed in the image. After testing the numerical data for normal distribution by using the Kolmogorov-Smirnov at a *p* < 0.0001, the hypothesis of not normally distributed data applies.

There was an excellent agreement of both readers for the average measurements for machine A (ICC = 0.958 (CI 0.938–0.9719)) and a moderate agreement for machine B (ICC = 0.679 (CI 0.535–0.778)).

**Fig. 5:**
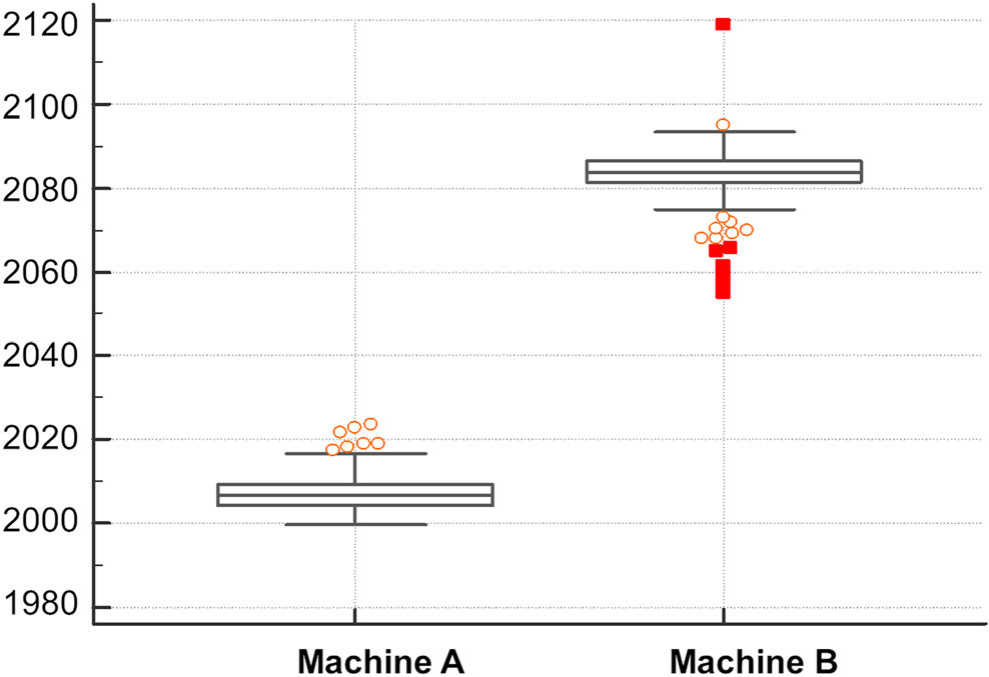
Boxplot of the comparison of the mean pixel values of the mammography images taken by mammography machine A and mammography machine B

**Fig. 6:**
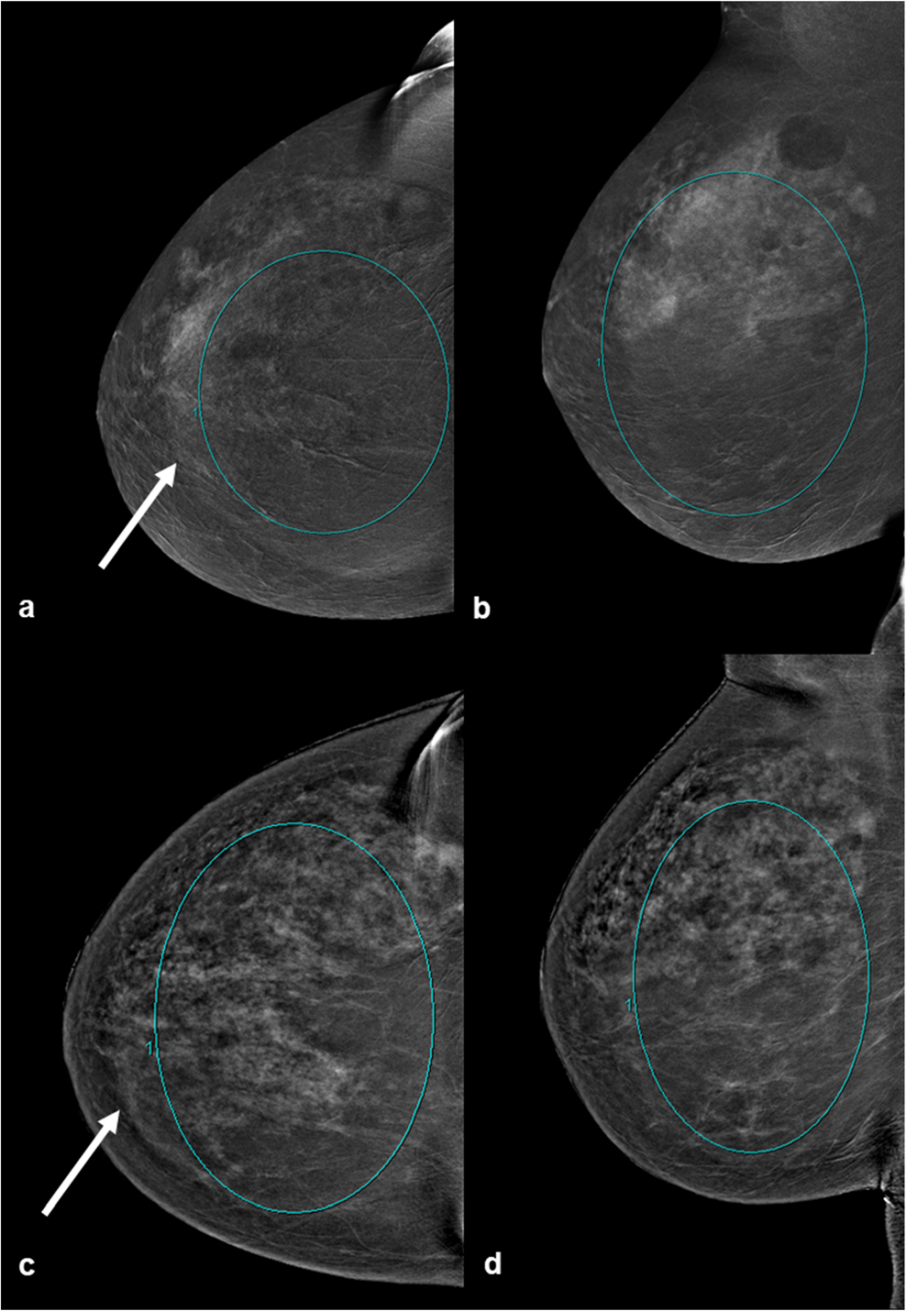
Comparison of the quantitative measurement of the background enhancement in the same woman with machine A (**a**, **b**) and machine B (**c**, **d**). Examinations were performed because of a high risk of breast cancer. In both cases iomoperol was used as contrast agent. The ROI was set to the most applicable extent, excluding the rim artifact in the images (arrow). Quantitative measured values with machine A were 2004.3 (**a**), 2018.7 (**b**) and those acquired with machine B were 2084.9 (**c**) and 2087.9 (**d**) respectively. Both examinations were correctly timed to the menstrual cycle and performed in a time span of 15 months

Comparing the mean values between the images of machine A and machine B showed a Hodges Lehmann median difference of a value of 76.70 in intensity enhancement. There was a significant difference between the quantitative overall background enhancement of the mammography machines compared in this study (*p* < 0.001). The background enhancement was lower for the cohort that was examined with machine A (Fig. 5). The evaluation showed a mean value of 2007 for machine A (IQR 2004.3 to 2009.3) and a value of 2083 for machine B (IQR (2083 to 2086.3). An example of the qualitative analysis is shown in Fig. 6.

### Impact of potential biases

The results of the multivariate regression analysis showed that the factor machine with a log odds of 147.74 was the only factor to have a significant impact on the background enhancement (*p* < 0.001). The factors contrast agent (*p* = 0.085; log odds = 1.072), breast density (*p* = 0.164; log odds = 0.784), age (*p* = 0.195; log odds = 0.710), and menopausal status (*p* = 0.313; log odds = 0.503) had no relevant impact on the quantitative background enhancement and therefore can be excluded as potential biases.

### Lesion conspicuity

The evaluation of the lesion conspicuity showed a significant difference between machine A and machine B, showing a significantly better lesion detectability for machine A (Tables 4 and 5) for the first (*p* = 0.009) and the second reader (*p* < 0.001).

**Table 4:** Results of the lesion conspicuity of both readers for machine A

Level of LC	R1 count	R1 percentage	R2 count	R2 percentage
1	1	0.031	1	0.031
2	2	0.062	2	0.062
3	7	0.219	4	0.125
4	10	0.313	13	0.406
5	12	0.375	12	0.375
Total	32	1.000	32	1.000
Median (IQR)	4 (3–5)		4 (4–5)	

*LC* lesion conspicuity, *R1* reader 1, *R2* reader 2

**Table 5:** Results of the lesion conspicuity of both readers for machine B

Level of LC	R1 count	R1 percentage	R2 count	R2 percentage
1	10	0.233	9	0.209
2	8	0.186	10	0.232
3	5	0.116	9	0.209
4	11	0.256	7	0.163
5	9	0.209	8	0.186
Total	43	1.000	43	1.000
Median (IQR)	3 (2–5)		2 (2–5)	

*LC* lesion conspicuity, *R1* reader 1, *R2* reader 2

## Discussion

In this study, we compared two different mammography machines regarding overall background enhancement in CESM images. Statistically significant differences in background enhancement could be observed using qualitative and quantitative measurement. Therefore, a possible impact on the image assessability has to be discussed.

There are few studies that have investigated the influence of background enhancement on sensitivity and specificity in CESM, showing similar results as found in MRI [7, 31, 32]. As shown in previous studies with reference to the background enhancement of MRI and CESM examinations, a higher background enhancement in both CESM and MRI could mask pathologic findings and consequently reduce diagnostic accuracy [7, 18, 20, 21, 31–33], especially since the evaluation in everyday clinical practice is largely performed qualitatively by the evaluating radiologist. Some factors have been proven to influence background enhancement in CESM. Similar to MRI, background enhancement is influenced by the status of the menstrual cycle [7]. Since particularly younger women who tend to have a higher breast density have a higher risk of developing multicentric and triple negative breast cancer, it is essential to ensure a high diagnostic imaging quality [34]. In case of a suspicion of cancer or biopsy-proven breast cancer, a timing of the examination to the menstrual cycle should not be performed in order to avoid a delay in diagnosis. Therefore, imaging methods ensuring a low background enhancement are essential. Our study could show that the parameters contrast agent, age, breast density, and menstrual cycle had no significant impact on background enhancement. At the same time, our results highly indicate a relevant impact of the mammography machine on the background enhancement. The fact that the findings of the qualitative analysis were concordant with the quantitative results allows the assumption that the difference is presumably recognizable for the diagnosing radiologist. As our results show a subjectively better delineation of the pathological findings on images of machine A, the lower background enhancement could be one of the reasons for the better detectability of pathological findings. In concordance with this assumption, the conspicuity of the pathology in CESM images that were acquired with machine A were significantly better than that in machine B. However, for the evaluation, the conventional mammogram was not considered. Thus, although the lesion conspicuity in CESM images differs, the final impact on the diagnostic confidence remains unclear.

In our study, the anode/filter combination, the film-focus distance, and the running grid of both machines differed. In addition, the images of machine A were acquired with 22–49 kv, while machine B used 20–49 kv. The subtraction of higher energy shots could reduce the overall background enhancement which might be one explanation of the continuous higher measurable background enhancement. The possible influence of the anode/filter combination and the post processing on artifacts in CESM images has already been shown [22]. Thus, a certain impact on the background enhancement is conceivable.

To the best of our knowledge, this is the first study to investigate the possible impact of the mammography machine on overall background enhancement. Therefore, further investigations on the cause of the difference of the overall background enhancement and its possible impact on the diagnostic accuracy are necessary. As we were not able to make changes to the devices used within the study, the different parameters and settings should be tested to see if they affect the enhancement.

Regarding our study, some limitations have to be considered. Although we proved a qualitative and quantitative difference between two mammography machines, it remains unclear whether this could lead to a relevant influence on diagnostic accuracy as there is a learning curve with different image impressions of different machines and vendors. The difference in lesion conspicuity could imply an influence on diagnostic accuracy. Furthermore, only two mammography devices were compared. Further studies are necessary to compare other mammography devices.

## Conclusion

The study shows a statistically significant difference in the qualitative and quantitative overall background enhancement between two mammography devices from different manufacturers.

## Supplementary information

ESM 1 (DOCX 17 kb)

## Abbreviations

CC: Craniocaudal view
CESM: Contrast-enhanced spectral mammography
ICC: Intraclass correlation coefficient
MLO: Mediolateral oblique
MRI: Magnetic resonance imaging
ROI: Region of interest

## Declarations

### Guarantor

The scientific guarantor of this publication is PD Dr. Heike Preibsch, MD.

### Statistics and biometry

No complex statistical methods were necessary for this paper.

### Methodology

• retrospective

• observational

• performed at one institution
