# Supplementary material for: Background enhancement in contrast-enhanced spectral mammography (CESM): are there qualitative and quantitative differences between imaging systems?
Source: Eur Radiol. 2022 Dec 6;33(4):2945–53. doi: 10.1007/s00330-022-09238-9 (PMC10017655; doi:10.1007/s00330-022-09238-9)
Supplement: Supplementary file 1 — (DOCX 17 kb) [file 330_2022_9238_MOESM1_ESM.docx]

**Supplementary material – Lesion assessment**

Regarding lesion conspicuity the number of lesions within the image was counted and the maximum lesion diameter within the image were documented. For evaluation, it was considered whether the lesion could be correctly analyzed as benignant or suspicious. Histopathology was used as reference. In one case, due to high background enhancement, the examination was evaluated as non-diagnostic so that lesions could not be counted or evaluated. The results are illustrated in tables 7-11.

**Supplementary material - Tables**

**Table 6: Distribution of the quantitative results of the second reader for machine A and B**

| **Percentage** |  | **Machine A** | **Machine B** |
| --- | --- | --- | --- |
| 100.0% | maximum | 2055 | 2099.36 |
| 99.5% |  | 2055 | 2099.36 |
| 97.5% |  | 2022.904 | 2090.822 |
| 90.0% |  | 2013.044 | 2088.04 |
| 75.0% | quartile | 2010.04 | 2086.36 |
| 50.0% | median | 2007.12 | 2083.84 |
| 25.0% | quartile | 2004.755 | 2080.4 |
| 10.0% |  | 2003.638 | 2076.634 |
| 2.5% |  | 2001.225 | 2065.502 |
| 0.5% |  | 2000.53 | 2028.11 |
| 0.0% | minimum | 2000.53 | 2028.11 |

**Table 7: Number of lesions counted for machine A**

| **Reader** | **Number of Lesions** | **Benignant lesions** | **Suspicious lesions** | **Non-diagnostic** |
| --- | --- | --- | --- | --- |
| Reader 1 | 68 | 10 | 58 | 0 |
| Reader 2 | 68 | 10 | 58 | 0 |

**Table 8: Number of lesions counted for machine B**

| **Reader** | **Number of Lesions** | **Benignant lesions** | **Suspicious lesions** | **Non-diagnostic** |
| --- | --- | --- | --- | --- |
| Reader 1 | 108 | 12 | 96 | 1 |
| Reader 2 | 110 | 12 | 97 | 1 |

**Table 9: Lesion assessment**

| Machine B Machine A |  |  |  |  | |
| --- | --- | --- | --- | --- | --- |
|  |  | **Reader 1** |  | **Reader 2** | |
|  |  |  |  |  |  |
|  |  |  | Count | Category | Count |
|  |  | Benign | 9 | Benign | 9 |
|  |  | False negative benign | 0 | False negative benign | 0 |
|  |  | Malignant | 58 | Malignant | 58 |
|  |  | False positive benign | 0 | False positive benign | 0 |
|  |  | False positive suspicious | 1 | False positive suspicious | 1 |
|  |  | Total | 68 | Total | 68 |
|  |  |  |  |  |  |
|  |  |  |  |  |  |
|  |  |  | Count | Category | Count |
|  |  | Benign | 10 | Benign | 10 |
|  |  | False negative benign | 0 | False negative benign | 0 |
|  |  | False positive benign | 2 | False positive benign | 3 |
|  |  | Malignant | 86 | Malignant | 86 |
|  |  | False positive suspicious | 10 | False positive suspicious | 11 |
|  |  | Total | 108 | Total | 110 |

**Table 10: Maximal lesion diameter machine A**

| **Percentage** |  | **Reader 1** | **Reader 2** |
| --- | --- | --- | --- |
| 100.0% | maximum | 116 | 117 |
| 99.5% |  | 116 | 117 |
| 97.5% |  | 115.2 | 116.2 |
| 90.0% |  | 71 | 71.8 |
| 75.0% | quartile | 39 | 42 |
| 50.0% | median | 23.5 | 24,5 |
| 25.0% | quartile | 13.25 | 14 |
| 10.0% |  | 8.1 | 8,1 |
| 2.5% |  | 3.025 | 3.025 |
| 0.5% |  | 3 | 3 |
| 0.0% | minimum | 3 | 3 |

**Table 11: Maximal lesion diameter machine B**

| **Percentage** |  | **Reader 1** | **Reader 2** |
| --- | --- | --- | --- |
| 100.0% | maximum | 88 | 92 |
| 99.5% |  | 88 | 92 |
| 97.5% |  | 88 | 92 |
| 90.0% |  | 63 | 62 |
| 75.0% | quartile | 31 | 33 |
| 50.0% | median | 19 | 20 |
| 25.0% | quartile | 14 | 14 |
| 10.0% |  | 9 | 9 |
| 2.5% |  | 6 | 6 |
| 0.5% |  | 6 | 6 |
| 0.0% | minimum | 6 | 6 |

**Supplementary material - Figures**

**Figure 7:**

Images of the same woman who underwent CESM twice in a range of 14 months because of a contralateral carcinoma. At both examinations, the patient was under systemic therapy. The images taken with machine A (a+b) show a lower background enhancement that was rated as mild while the background enhancement of machine B (c+d) was rated as moderate by both readers.
